# A randomised, double-blind, placebo-controlled clinical trial assessing the efficacy of bedtime buddy® for the treatment of nocturnal enuresis in children

**DOI:** 10.1186/s12887-019-1797-8

**Published:** 2019-11-09

**Authors:** Janet Schloss, Kimberley Ryan, Rebecca Reid, Amie Steel

**Affiliations:** 1 Office of Research, Endeavour College of Natural Health, Brisbane, Australia; 2 Australian Research Centre in Complementary and Integrative Medicine, Faculty of Health, University of Technology Sydney, Sydney, Australia

**Keywords:** Nocturnal enuresis, Bladder incontinence, Herbal medicine

## Abstract

**Background:**

Nocturnal enuresis (NE), or ‘bedwetting’, is a form of night-time urinary incontinence occurring in younger children. A diagnosis of NE can be socially disruptive and psychologically stressful for a child. The most common strategies used by parents to manage NE are waking the child during the night to use the bathroom and limiting the child’s water intake before going to bed. Behavioural or educational therapies for NE such as urotherapy or bladder retraining are widely accepted and considered as a mainstream treatment option for non-neurogenic lower urinary tract dysfunction in children. Pharmacotherapy also plays an ancillary role. However, there is no gold standard therapy or intervention to effectively manage NE.

**Methods:**

This study aims to determine the efficacy of a herbal combination in the treatment of NE in children. The target population for this study is 80 children aged between 6 and 14 years old (males and females) who have primary nocturnal enuresis ≥3 per week (wet nights). The active group will receive one or two capsules per day containing 420 mg of a proprietary blend of Urox® (Seipel Group, Brisbane, Australia) containing Cratevox™ (*Crataeva nurvala* L; Capparidaceae; Varuna) stem bark extract standardised for 1.5% lupeol: non-standardised *Equisetum arvense* L. (Equisetaceae; Horsetail) stem extract; and, non-standardised *Lindera aggregata* Sims. The primary outcome for this study is the frequency of nocturia. Secondary outcomes include safety, quality of life, and daytime incontinence. Each participation will be involved in the trial for 32 weeks including contact with the research team every 2 weeks for the first 8 weeks and then every 8 weeks until trial completion.

**Discussion:**

This study examines a novel treatment for an under-researched health condition affecting many children. Despite the availability of several therapies for NE, there is insufficient evidence to support the use of any one intervention and as such this randomised placebo-controlled phase II trial will be an important contribution to understanding potential new treatments for this condition.

**Trial registration:**

Australian and New Zealand Clinical Trials Registration Number: 12618000288224.

Protocol: 23 February 2018, version 1.1.

## Background

Nocturnal enuresis (NE), or ‘bedwetting’, is a form of night time urinary incontinence occurring in younger children [1, 2]. More specifically, primary nocturnal enuresis (PNE) is defined as the involuntary discharge of urine at night in children aged 5 years or older, who do not have congenital or acquired defects of the central nervous system or urinary tract and have not experienced a dry period of more than 6 months [1]. NE is classified as either *monosymptomatic enuresis* – enuresis with no other lower urinary tract symptoms – or *non-monosymptomatic enuresis* – enuresis with other, mainly daytime, lower urinary tract symptoms [3]. The aetiology of NE has been attributed to multifactorial pathophysiological factors such as an altered antidiuretic hormone profile with nocturnal polyuria, arousal failure, delayed bladder maturation and nocturnal detrusor overactivity [4, 5]. Common co-morbidities identified with NE are: constipation, diabetes mellitus, developmental attention or learning difficulties, history of recurrent urinary tract infection (UTI) in boys, and pinworm infestation [6–8]. Current NE international prevalence rates are reported as between 5 and 20% of children over 5 years old [4, 7, 9–11] with an incidence as high as 18.2% in an Australian school-aged children cohort. Predictive or risk factors which have been linked with NE include: age, male gender, daytime incontinence, encopresis, social concerns, delayed age of walking, positive parental history of enuresis, and sibling history of enuresis [7, 9, 12].

A diagnosis of NE can be socially disruptive and psychologically stressful for a child [2, 10]. Quality of life indicators have been explored in the past to determine the impact of NE on the life of children [9, 13, 14]. The psychological impact of bladder dysfunction has been understood to be due to the urinary symptoms themselves [13]. Though not a causal factor, poor school performance has been reported to be higher in children with NE [9]. A child’s interpretation of the impact of NE on their own life also appears to vary from the parental proxy perception of quality of life [13, 15]. Parents have reported more often (71%) that NE had little or no impact on their child’s overall quality of life, as they believed it was a developmental condition [9]. This is supported by the fact that only half the amount of parents consult a medical practitioner for this condition [16], which suggests that the prevalence could be higher in the community than indicated by current documented Figures [17].

### Common approaches to treatment for nocturnal enuresis

Parents often choose a combination of therapies to treat their children with NE. The most common strategies utilised are waking the child during the night to use the bathroom and limiting the child’s water intake before going to bed [18]. Behavioural or educational therapies for NE such as urotherapy or bladder retraining are widely accepted and considered as a mainstream treatment option for non-neurogenic lower urinary tract dysfunction in children [19] with the aim to normalise bladder emptying and storage by teaching relaxed voiding techniques [20]. Alarm interventions have demonstrated moderate effectiveness in reducing NE in approximately two thirds of children with relapse occurring in half children with ceasing the treatment [21]. Transcutaneous electrical nerve stimulation (TENS) has been examined for the treatment of NE, but has mixed efficacy, with reported benefit in reducing NE events in children with primary NE [22] yet no effect on children with monosymptomatic NE [23].

Pharmacotherapy plays an ancillary role with generally accepted pharmacological treatment options such as desmopressin, tricyclics, or anticholinergics administered orally [6]. Though much research has focussed on using pharmacotherapy to treat NE, only a minority of children actually receive such medication (9.1%) [18]. Most children prescribed medications such as tricyclics and desmopressin have shown improvement in reducing the number of wet nights but have been shown to relapse after stopping active treatment [21]. Parents have also demonstrated low compliance (17.2%) in persisting with medication for their children with NE [18].

The adverse effects of desmopressin are dose related [24] with most reactions related to study medication manifesting as drug hypersensitivity symptoms such as; abdominal pain, headache, nausea, flushing face, and asthma-related symptoms. An integrative approach to treatment has been suggested in the past, given the multifactorial nature of NE, issues of pharmacotherapy treatment refractoriness, and considerations to risk/benefit in treatments [1]. NE has the potential to significantly impact the psychological and social wellbeing of a child, thus alternative treatment options that could be administered with reduced adverse effects need to be explored. However, without an established standard treatment with strong evidence effectiveness, new treatments should be examined through placebo-controlled studies [25].

### Herbal medicine for nocturnal enuresis

Given that prescription medications are cautiously used in children and potentially have undesirable side effect profiles, controlled clinical trials are needed to explore complementary medicine (CM) treatments such as herbal medicine for safety and efficacy. A Cochrane review on complementary medicine and miscellaneous interventions for NE was published in 2011 and highlighted the need for larger and more rigorous trials examining CM treatment options [10]. The review concluded that although the outcome measures varied, herbal medicine seemed to perform better than imipramine, both during and after treatment [10].

Herbal medicine has traditionally been used in the treatment of symptoms for NE or urinary incontinence [10, 11]. A historical account by Glicklich [26] chronicles the use of herbal medicine to treat children with enuresis from as early as the time of the Papyrus Ebers dated 1550 B.C. In contemporary society, parents of children with NE frequently seek herbal products from pharmacists to treat this condition, which has led to an increased consumer demand and an associated increased availability in pharmacies [27]. Prior and emerging clinical research into the use of herbal medicine continues to show promising outcomes and increased benefits in reducing the relapse of symptoms in NE [28, 29]. Early herbal medicine research for the treatment of NE has mainly utilised herbs drawn from Traditional Chinese medicine practices [1, 11].

### UROX® and adult urinary incontinence

**Fig. 1:**
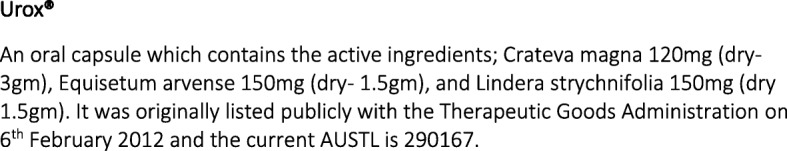
Summary of formulation information

Urox® is an herbal formulation listed with the Australian Therapeutic Goods Administration which contains a combination of *Crateva nurvala*, *Equisetum arvense* and *Lindera aggregate* (see Fig. 1). Urox® has been trialled previously in a phase 2, randomised, double-blind placebo controlled trial to reduce the symptoms of overactive bladder (OAB) and urinary incontinence (UI) in adults (NCT02396160) [30]. One hundred and fifty patients were randomised to receive active or placebo capsules for 8 weeks. The primary outcome measure was urinary frequency defined as the number of voluntary diurnal and / or nocturnal micturition, self-reported via a validated urinary diary. Secondary outcomes included the common urinary symptoms of; urinary frequency, nocturia, urgency and stress incontinence using participant micturition diaries. Further secondary outcomes included health-related quality of life measurements using validated questionnaires, the incontinence impact questionnaire (IIQ) and the urinary distress inventory (UDI) [30].

The study determined that there was a reduction in all urinary symptoms (i.e. day frequency, nocturia, urgency, urgency incontinence, stress incontinence and total incontinence) which reached statistical significance in all parameters. Specifically, day frequency (n/day) reduced from 11.53 ± .54 to 7.69 ± 2.15, statistically significant when treatment and placebo groups were compared (*p* = 0.01, 0.01–0.02). The frequency of nocturia (n/day) was also notably reduced 4.02 ± 1.62 to 2.16 ± 1.49 after 8 weeks of treatment. The treatment vs placebo at 8 weeks was statistically and clinically significant (*p* = 0.03, 0.02–0.05). Urinary urgency (OR 0.02; 95%CI 0.01 to 0.03), and total incontinence (OR 0.03; 95% CI 0.01 to 0.06) were also lower (all *p* < 0.01) in the treatment group. Quality of life was also noted to be significantly improved after treatment in comparison to placebo utilising [30].

## Methods/design

### Objectives

#### Aim

The aim of the study is to determine the efficacy of the combination of *Crateva nurvala*, *Equisetum arvense* and *Lindera aggregata* in the treatment of nocturnal enuresis in children, as assessed by frequency of nocturnal enuresis.

#### Hypothesis

The combination capsule of the herbal formula ‘Urox- Bedtime Buddy’ will reduce the frequency of nocturnal enuresis in children aged 6 to 14 years.

#### Objectives

1. To improve the quality of life for children that experience bed wetting issues by reducing the number of times the child wets the bed a week.
2. To reduce the frequency of bed wetting a child experiences a week which has the potential to improve their physical, psychological and social wellbeing.

##### Trial design

This is a randomised, prospective, double-blind randomised, placebo-controlled trial in which participants, researchers and statisticians analysing the data are blinded to which group the participants are randomised. It is a parallel group or non-crossover study. The allocation ratio is 1:1.

### Methods: participants, interventions, and outcomes

#### Study setting

The research setting is Endeavour College of Natural Health, 269 Wickham Street, Fortitude Valley in Brisbane. There is a site agreement between UTS and Endeavour College of Natural Health, Brisbane Campus.

#### Eligibility criteria

The target population for this study is children aged between 6 and 14 years old (males and females) who have primary nocturnal enuresis ≥3 per week (wet nights). Potential participants will be excluded if they report a diagnosis of vesicoureteral reflux, spinal dysraphism, recent urotherapy, recent or current urinary tract infection (< 1 week), active cancer with current treatment, a history of cardiac failure and a history of renal disease or urinary retention.

#### Interventions

##### Active intervention

Each capsule contains 420 mg of a proprietary blend of Urox® (Seipel Group, Brisbane, Australia) containing Cratevox™ (*Crataeva nurvala* L; Capparidaceae; Varuna) stem bark extract standardised for 1.5% lupeol: non-standardised *Equisetum arvense* L. (Equisetaceae; Horsetail) stem extract; and, non-standardised *Lindera aggregata* Sims. The TGA Approval number for the product is – AUSTL 194355. Capsules were manufactured in a Therapeutic Goods Administration licensed facility according to the PIC/S Guide to Good Manufacturing Practice for Medicinal Products, PE 009–9-15 January 2009. Capsules were subjected to microbiological and heavy metal testing to ensure they complied with product specifications.

The adult dose is two capsules per day taken once daily with food. The dosage is 1 capsule in the morning (< 40 kg) or 2 capsules in the morning (> 40 kg) for children. The dosage regimen was determined based on several integral methods as follows; earlier research with *Crataeva* and *Equisetum* alone [31, 32] and more recently with Schoendorfer, et al’s trial [30] with Urox which includes the newer formulation with 3 ingredients; pharmacopeia and traditional herbal medicine textbook dosage recommendations [33, 34] and Clark’s rule for dosage in Children (Dose = Adult dose x Weight(kg) / 70) which is conservative and tends to underestimate the required dose [35]. Urox was listed on the ARTG 6th February, 2012 and was marketed in Australia from this date. The Bedtime Buddy is the same formula as Urox but dosed for children and was listed with the TGA on the 14th June, 2017 – AUSTL 290162.

##### Placebo

The active intervention will be compared with an identical vegetarian capsule containing colour-matched cellulose to that of the active ingredient. Participants will be prescribed 1 capsule in the morning (< 40 kg) or 2 capsules in the morning (> 40 kg).

#### Outcomes

##### Primary outcome

**Frequency of nocturnal enuresis (Week 8)**

The primary outcome for this study will measure efficacy as quantified by the change from baseline in the frequency of nocturnal enuresis at week 8. Parents are completing a daily diary (see Fig. 3) online or via hard copy which measures episodes of nocturia (waking wet through the night, and / or waking wet in the morning). The diary also captures the volume of fluid consumed in the day, together with the compliance of taking the capsule each day.

##### Secondary outcomes

**Frequency of nocturnal enuresis (Week 4 and Week 32)**

In addition to the primary outcome, change from baseline in the frequency of nocturnal enuresis at week 4 and Week 32 will also be measured.

**Safety (Week 4 and Week 8)**

Secondary outcomes in Stage 1 will include on the safety of the herbal formulation in children aged 6–14 years at Weeks 4 and 8 of the study. Data will be collected from adverse reactions (type, severity, relation to investigational product), and side effects experienced by the participant recorded in the diary. An interim analysis will be conducted after enrolment of 30 participants to assess safety and continuation of the trial. Also at this time point, a power calculation to confirm participant numbers needed to reach statistical significance will determine the total number of participants required for recruitment. Safety data will be continued to be collected throughout Stage 2.

**Quality of Life (Week 4, Week and Week 32)**

Further secondary outcomes will measure health-related quality of life as measured by the Paediatric quality-of-life questionnaire (PinQ). This is a well-validated tool that measures the impact of NE on paediatric quality of life [13]. Permission has been granted by the author to use in this trial [36]. The PinQ will be completed by parent and child on separate data collection forms as it has been shown that there has been inconsistencies in proxy and child answers when filling in the questionnaire [13]. If a child is unable to complete a questionnaire due to lack of cognitive or communicative development, the researcher will assist the child in developmentally appropriate language to complete the questions. The PinQ will be completed at Baseline, Week 4, Week 8 and Week 32. Change in Quality of Life will be calculated based on difference from baseline at Week 4, Week 8 and Week 32.

**Daytime incontinence symptoms (Week 4, Week 8 and Week 32)**

There is a possibility that the capsule will impact on day-time symptoms of incontinence in children as this was demonstrated in an adult clinical trial with the same formulation [30]. These symptoms are measured at baseline, Week 4, Week 8 (in clinic visits) and Week 16 and Week 24 (over the phone), and in the final visit at Week 32. Data collection will note if there has been episodes of day-time urinary incontinence, urgency, constipation, faecal incontinence. If the parent answers yes to any of the above categories, they average how many times per week it has occurred for each symptom. Change in daytime incontinence symptoms will measured as difference from baseline at week 8 and week 32.

**Total response to treatment (Week 4, Week 8 and Week 32)**

Response to treatment will also be calculated based on the International Children’s Continence Society guidelines [37]. Less than 50% reduction in wet nights will be defined as ‘no response’, 50–99% reduction in wet nights will be considered a ‘partial response’. Participants will only be defined as having a ‘response’ if a 100% reduction in wet nights is reported. This will be calculated based on difference from baseline at Weeks 4, 8 and 32.

#### Trial duration

**Fig. 2:**
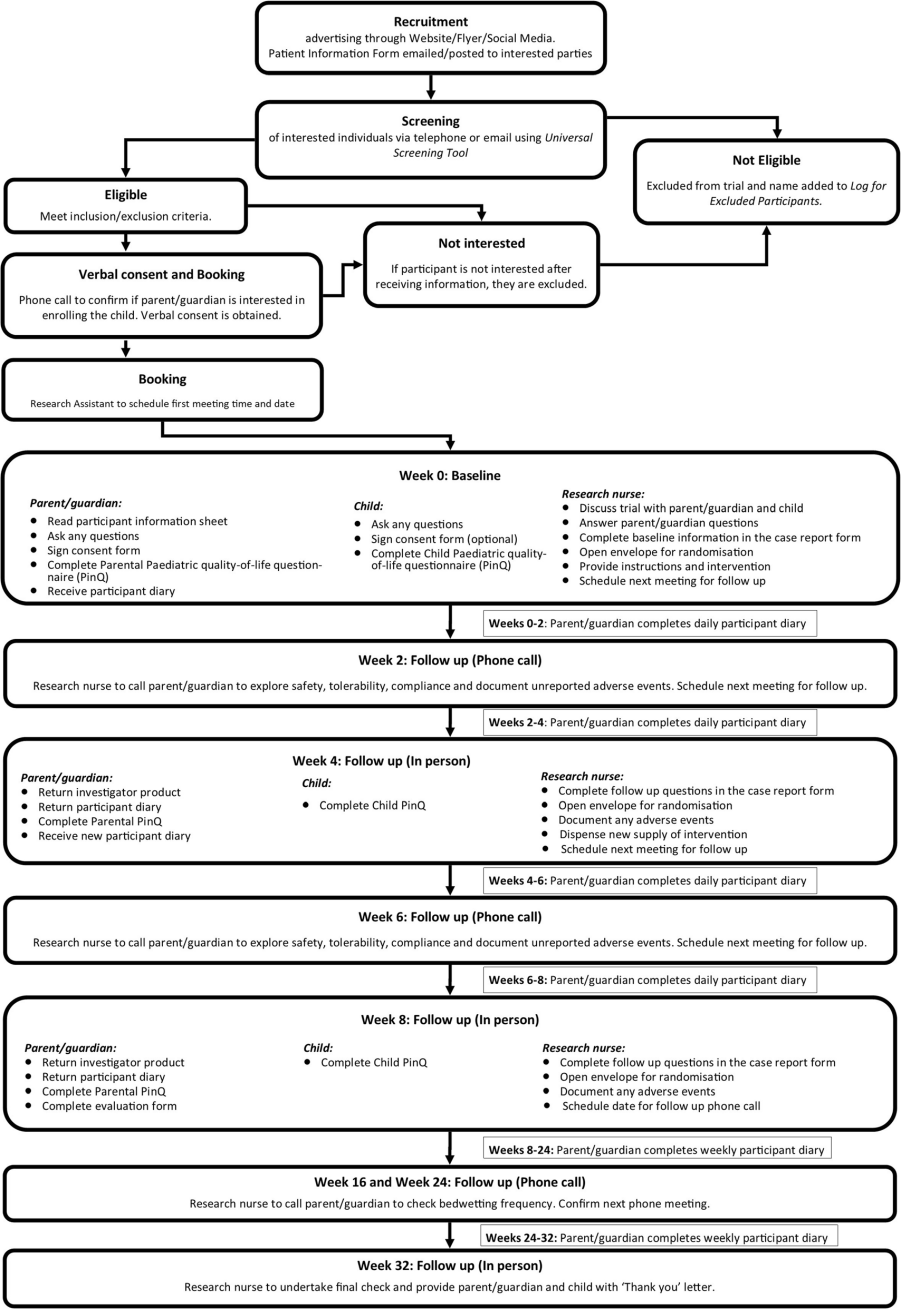
CONSORT flow chart

Each participation will be involved in the trial for 32 weeks. The overall flow of study involvement is reported in Fig. 2. The first 8 weeks involve the oral ingestion of the daily capsule. The post-interventional period involves weekly entry in the electronic diary recording any ongoing episodes of nocturia for 6 months to determine the long-term effects of the capsule on NE. There is also 2 monthly follow up phone calls to the note any new changes to medications or additional NE treatments as well as the daytime continence questions. A final visit to the clinic will occur at Week 32.

#### Sample size

To detect a difference between the intervention group and the control group, a calculation from a trial on desmopressin and oxybutynin [38] was conducted. The power calculation used 80% power for 5% significance based on three-month results (*p* = 0.001). This resulted in the number of children required to recruit being 80 participants. This allows for a 20% attrition rate which provides an adequate number of participants in the primary analysis.

Due to the absence of existing studies involving similar populations and interventions, an interim sample size calculation will be conducted to confirm and potentially readjust the sample size as appropriate. The recalculation of the sample size will be conducted after 30 participants have completed the trial. The difference in the change (from baseline) in the frequency of nocturnal enuresis at week 8 will be calculated. This difference will then be imputed into the same equation used for the sample size calculation applied during study design. Any readjustment on the trial recruitment numbers will be resubmitted to the authorising HREC committee with the interim sample size calculation.

#### Recruitment

Convenience sampling techniques will be used to target the wider social community of parents with children that are experiencing NE. Participants will be recruited from the general population through social media other general recruitment channels. Several strategies will be utilised in the recruitment including magazine advertisements, web page, social media (Twitter and Facebook), primary school newsletters, distribution of advertising flyers placed in a variety of locations (i.e. Wellnation Clinics reception, health food stores and holistic CM clinics in Brisbane). Interested individuals will be directed to contact the Researchers directly via phone or email to express interest in the trial. A participant information sheet is available to download on the trial webpage but also is emailed to potential participants on enquiry.

### Methods: assignment of interventions

A block randomisation allocation was used based on 10 blocks of eight with an allocation of 1:1. Two extra blocks of eight were generated for over 80 allocations.

#### Allocation

##### Sequence generation

A computer-generated number randomisation was utilised: https://www.random.org/. No stratification was implemented due to the specified age restriction of ages 6–14. The block randomisation sequence generated is provided in a separate document that is unavailable to those who enrol participants or assign interventions.

##### Allocation concealment mechanism

The allocation concealment is through the sealed envelope system to conceal the sequence until the interventions are assigned. The sequence is generated and sealed by the PI. All investigators are blinded to the allocation.

##### Implementation

The allocation sequence will be generated by the PI (JS). Participants are screened for eligibility over the phone prior to the baseline visit. At the baseline visit, informed consent is obtained from the child and parent by the Trial Co-ordinator. During the baseline visit, participants will be allocated a study number and a participant code. The study number is a number assigned at the time of enrolment and will be in chronological order, for e.g. 001, 002, 003. The participant will also be allocated a participant code which will be a 2 or 3 alphabetical letter sequence which corresponds with their first letter of the first, middle and last names. The participant will be allocated to their assigned interventional group at this stage and receive either the Active treatment or Placebo.

#### Blinding (masking)

Masking**:** All participants, care providers/parents, all investigators, and outcomes assessors will be blinded. All genders will be included. Unbinding of the allocation will not occur until the analysis is complete.

#### Adherence

Adherence to the protocol will be measured by the number of capsules remaining at Week 8, as counted by the researcher. No participants will be excluded based on incomplete adherence to the protocol. Sensitivity analyses to control for variations in adherence will be controlled for in the analysis. Participants that seek other treatment at any stage during their involvement in the trial will be classified as treatment failures in the analysis.

### Methods: data collection, management, and analysis

#### Data collection methods

The data will be collected by KR - a qualified Naturopath (5 years) and clinical registered nurse (RN) with experience in clinical trials and emergency medicine in a major tertiary hospital. She also holds a position as a Research Assistant in the Clinical Trials section of the Office of Research, Endeavour College of Natural Health and has 10 years clinical research experience on co-ordinating a variety of different trial designs. Data collection will occur at Weeks 0, 2, 4, 6, 8, 16, 24, and 32 by KR. Additionally data is collected daily for 8 weeks via parental electronic diary and then weekly for 6 months.

#### Screening

Once a parent, guardian or child has shown interest in the trial via contacting the researchers, an explanation of the clinical trial and what is involved in participation initially occurs over the phone. The researcher will email, post, or direct the parent to the clinical trial website where they can download a copy of the Participant Information Sheet to read independently. The parent will be encouraged to discuss the participation with the child themselves and someone else who is able to support them in making their decision.

A follow up phone call or email will occur several days to a week later to verify the parent has read the information and discussed it with their child. This is an opportunity for further questions by the parent if needed. During this phone call the researcher will ask the parent if they would like their child to participate in the trial. If the parent says yes, the researcher will use the screening tool i.e. Participant screening procedure form (Universal), to determine eligibility in accordance with the defined eligibility criteria. All eligibility details collected through the screening process and before enrolment will be based on parent report of existing diagnoses rather than evaluation from an urologist or paediatrician. If the child is eligible an appointment date and time will be created for the participant and parent to attend the clinical trial site.

#### Assessments

Outcomes will be assessed by a *Case Report Form*, the *Paediatric Quality-of-Life Questionnaire (PinQ)*, and a *Participant Diary*.

##### Case report form

The Case Report Form (CRF) will be completed by the researcher at weeks 0, 2, 4, 6, 8, 16, 24, and 32. The baseline data collection will include demographic data, height and weight measurement, medications including complementary and over the counter medicines they are taking, medical history, risk factors for NE, prior treatments for NE, continence history, fluid intake per day. There is ongoing data collection fortnightly of any adverse events or concomitant medication via phone call or clinic visit whilst taking the intervention. As part of the CRF, a quality of life questionnaire (PinQ) will be completed by both the participant and parent/guardian at baseline, week 4, 8 and 32. The quality of life questionnaire chosen is the Paediatric Quality of Life Questionnaire (PinQ).

##### Paediatric quality-of-life questionnaire (PinQ)

The impact of NE on the child’s quality of life will be measured by the PinQ (Paediatric quality-of-life questionnaire) [13]. The PinQ is a tool used to measure urinary incontinence Quality of Life in children with voiding dysfunction. It has been reliably tested and validated in numerous trials since its conception [2, 14, 39]. Permission has been granted from the author for its use in this trial.

The PinQ will be completed by both parent and child on separate data collection forms as it has been shown that there has been inconsistencies in proxy and child answers when filling in the questionnaire [13]. It is completed by both parent and child as prior research has highlighted differences in reporting with proxy and self-reporting in the same questionnaire [14]. Children as young as 5 years old have been shown to reliably and validly self-report on their health-related quality of life in questionnaires when given the opportunity with an age-appropriate instrument [40]. If a child is unable to complete a questionnaire due to lack of cognitive or communicative development, the researcher will assist the child in developmentally appropriate language to complete the questions rather than the parent influence the response. The PinQ will be completed by both parent and child at Baseline, Week 4, Week 8 and finally in Week 32.

##### Diary

The daily diary for the first 8 weeks will constitute questions covering three main areas: compliance, fluid intake, and continence. There will be the option for the participant to complete it as an electronic diary or as a paper based one. This is for ease of the participant in their own environment. In the case of the electronic diary, the researcher will set up a widget on the parent’s smartphone. This is a link that takes the participant directly to their individual eDiary (which is identical to the paper one) where the parent is required to fill it in daily. Each participant will have an individual link which is linked to their own data within the online survey platform (Survey Gizmo). From week 9 to week 32, a weekly diary entry instead of daily will be required to record any weekly events of nocturia. This will be made available as an eDiary or a paper-based diary depending on the parent/guardian.

#### Data management

All participant hard copy files which include; CRF, Consent Form, and PinQ diaries will be kept in individual files in plastic sleeves in a locked filing cabinet. The filing cabinet is inside the locked Clinical Trials Office, which is located in the department of the Office of Research, Level 4, Endeavour College of Natural Health. The only people accessible to this office and filing cabinet are the Clinical Trial Co-ordinator (JS) and Research Assistant (KR). Electronic forms and logs will be maintained as password protected files on the computers of the researchers which are only accessible by the researchers named. This includes the following documents; Participant screening procedure form (Universal), Screening log for excluded patients and the Patient identification and enrolment log. The eDiary information is stored on a password protected survey platform (Survey gizmo). Data access is limited only to the research team assigned to that project. The hard copy data files will be stored in a locked filing cabinet in the Researcher’s office for 7 years, then destroyed permanently. The electronic data logs will be archived under password protected files on local drive for 7 years from commencement date of trial.

#### Statistical methods

All data will be analysed on STATA©14. Analyses will be conducted on an intention to treat basis. Descriptive and inferential statistics will be conducted. The difference in change between groups will be analysed using paired T-tests and Analysis of Variance (ANOVA). Groups will be compared at baseline and if difference(s) is/are ascertained, the variable(s) will be entered into the regression model to appropriately adjust the analyses. Sensitivity analyses will also be conducted to account for incomplete adherence to protocol. Interim statistical analysis for safety will occur after 30 participants have completed. This is same timing as the re-evaluation of the sample size calculation. All baseline, demographic, safety and efficacy outputs data will be summarised by treatment group. In summary tables of continuous variables, the minimum and maximum statistics the arithmetic mean and median, the 95% confidence interval, standard deviation, and standard error will be presented to the same number of decimal places as the original data. Safety outputs will be documented at the interim analysis and end of trial in columns with number, severity, treatment group and percentage. All hypothesis testing will be carried out at the 5% (2-sided) significance level unless otherwise specified. *P* values will be rounded to three decimal places. *P* values less than 0.001 will be reported as < 0.001 in tables (Table 1).

**Table 1:** Details of the active and control interventions

Arms	Assigned Interventions
Active Comparator: Treatment 1 capsule in the morning (≤40 kg) or 2 capsules in the morning (> 40 kg) for 8 weeks duration.	Dietary supplement: Bedtime Buddy Active treatment- Proprietary combination of 420 mg of herbs. expressed as dry herb equivalent: Crateva nurvala 3000 mg (120 mg) *Equisetum arvense* 1500 mg (150 mg) Lindera Aggregata 1500 mg (150 mg)
Control Comparator: Placebo 1 capsule in the morning (≤40 kg) or 2 capsules in the morning (> 40 kg) for 8 weeks duration.	Placebo- Identical vegetarian capsule containing colour-matched cellulose to that of the active treatment

### Methods: participant timeline

**Fig. 3:**
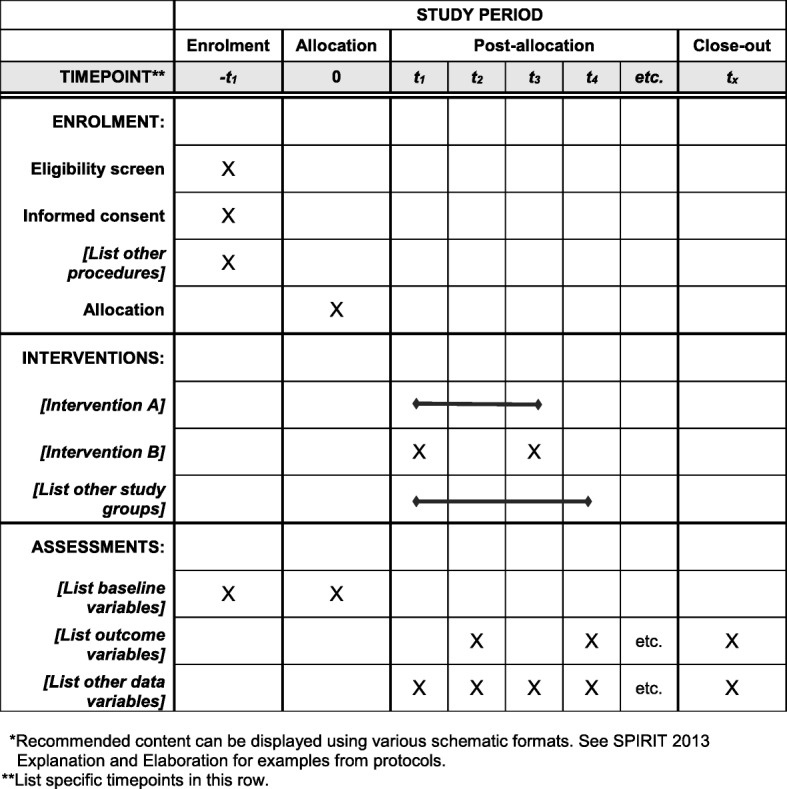
Schedule of enrolment, interventions, and assessments

Details for the schedule of enrolment, interventions and assessments of this study is depicted in Fig. 3.

### Week 0 (visit 1)

This is the initial baseline visit in which the parent and child are both present. It will take approximately 1 hour. After the researcher has outlined the trial and the potential risks and the parent and child both understand what participation involves, informed consent is obtained. At the baseline visit, a CRF will be firstly be completed by researcher in consultation with the parent. The parent and child will both independently complete the PinQ questionnaire at separate desks. The researcher will then explain the eDiary to the parent and help them save the eDiary link on their smartphone and give them a paper version if they are unable to use their phone. The next visit will be scheduled for 4 weeks’ time (Week 4) and will be booked at this appointment. The researcher will contact the parent after 1–2 days of commencing the intervention. This is over the phone or email to assess the participant for any initial potential adverse or allergic reaction.

### Week 2 (phone call only)

The researcher will call the parent to check adherence with the intervention and the diary as well as adverse reactions and changes to concomitant medications.

### Week 4 (visit 2)

The first bottle of capsules are returned at this visit to allow the researcher to count and record remaining intervention for confirmation of adherence. Those parents who chose to complete the paper diary will also return the completed diary. Any adverse events and concomitant medications will be noted and actioned if required. The PinQ will be completed by the parent and the child and the researcher will collect day-time continence data about the impact of the capsule on any day symptoms. Another 4 weeks supply of the capsules will be dispensed to the participant. It is anticipated this visit will take 30 min. The next visit will be scheduled for another 4 weeks (Week 8).

### Week 6 (phone call only)

The RA will call the parent to check adherence with the intervention and the diary as well as adverse reactions and changes to concomitant medications.

### Week 8 (visit 3)

The child and parent will attend this final visit to deliver the remaining bottle of capsules and diary. The RA will count and record any remaining intervention. Any adverse events and concomitant medications will be noted and actioned if required. The PinQ will be completed by the parent and the child and the RA will collect data about day continence. They will be offered paper diaries to cover the period from Week 8 to Week 32 if they do not wish to use the ediary format, with a pre-paid envelope to send them back to the college at the end of the trial. The visit is anticipated to take 30 min.

### Weeks 16 and 24 (phone call only)

The RA will contact the parent to check for any adverse reactions or concomitant medications, ask follow up questions for day time continence, and check diary compliance.

### Week 32 (visit)

The parent and child will attend the college for one final visit. The RA will finally check for any adverse reactions and concomitant medications, ask follow up questions for day time continence, and check diary compliance. The parent and child will complete the PinQ for a final time and paper diaries submitted if necessary.

### Trial completion

The trial is completed for the participant and they will receive a thank you letter for their participation. If they have indicated on their consent form that they wish to receive a copy of the Summary of Findings, their name will be added to a list and they will be contacted when the findings have been published.

### Monitoring

Minimal side effects were noted from the Urox trial in adults, with two episodes of diarrhoea, two urinary tract infections and one episode of flatulence. There were numerous side effects in the Placebo group; urinary tract infection, diarrhoea, headache worsened asthma, worsened memory, facial flushing, halitosis and worsened arthritic pain [30]. The Safety Summary for the product is available upon request. Safety data on side effects and adverse events associated with the intervention will be closely monitored and reported.

**Fig. 4:**
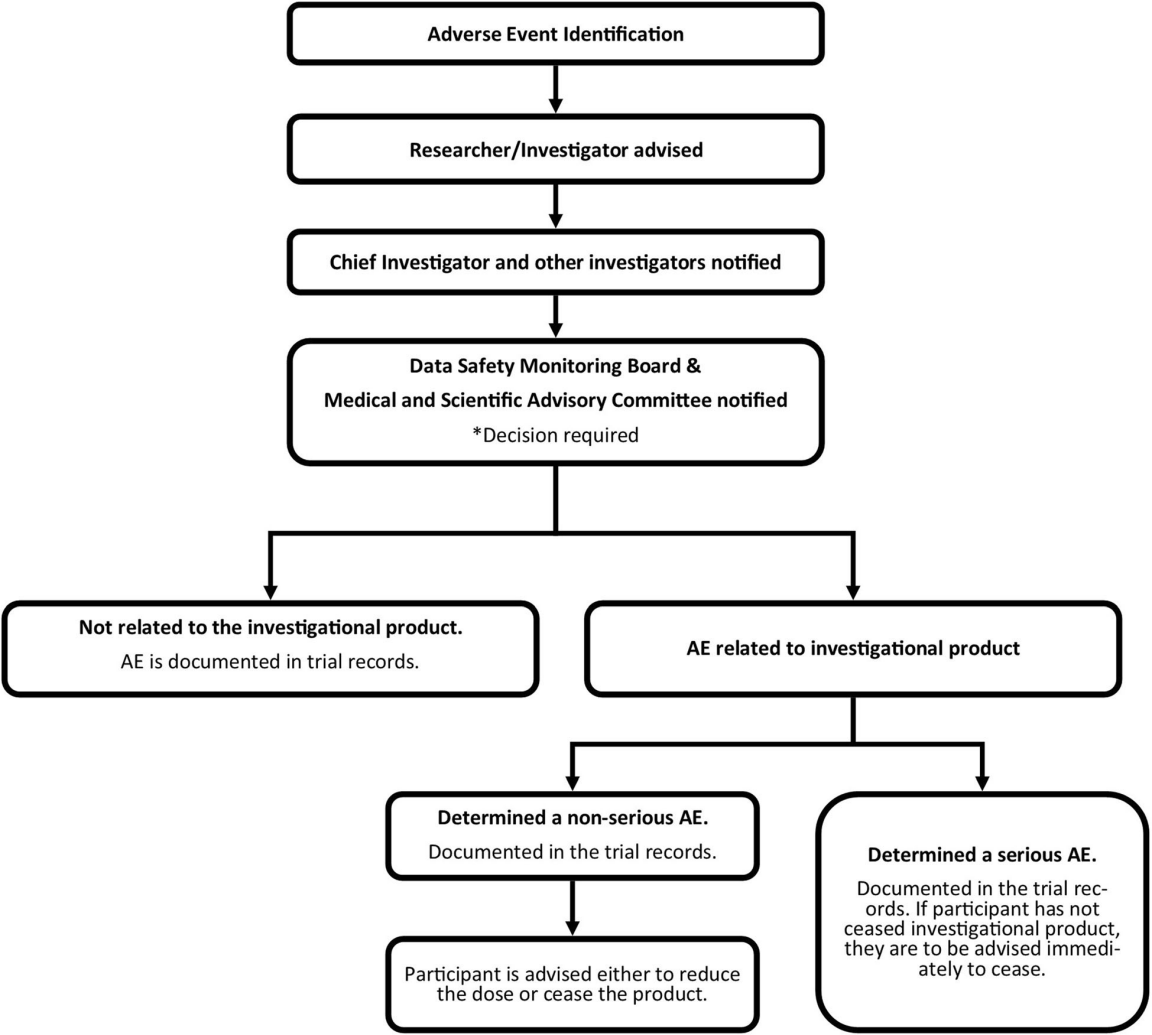
Adverse event flow chart

The Patient Information Sheet outlines that the parent / carer is able to phone the Researcher at any time if they feel that the child is experiencing an adverse reaction to the capsule. Parents are contacted via phone at 1–2 days after commencement of the capsule to determine tolerability and assess for potential adverse reactions. The electronic diary also provides parents with an opportunity to note any potential episodes of the child feeling unwell with an option to request the researchers contact them to further follow up. The researcher will determine if the reaction is an adverse event (AE) or a serious adverse event (SAE) and follow the adverse event flow chart (see Fig. 4). All AEs are also noted in the Adverse Events Log and individual participant case report form.

A Safety Advisory Board overseeing any serious adverse events (SAE) consists of three independent expert representatives (General Practitioner, Pharmacist and Herbal Medicine researcher and scholar). Any SAE’s will assessed by the safety committee with 24 h to determine if the adverse event is attributed to the interventional agent. The ensuing action will be decided between the Safety Advisory Board and the investigators.

In light of new information about the treatment that may arise during the trial, the participant / parent will be notified and it will be discussed with the parent whether they want the child / participant to continue on the trial. If it is decided that the participant will continue in the study, they will be asked to sign an updated consent form. Also, on receiving new information on potential harms, the researchers might consider it appropriate to withdraw specific a participant from the clinical trial. If this happens, the researcher will explain the reasons and arrange for the participant’s regular health care to continue by sending an updated letter to the child’s local medical practitioner.

An interim analysis will be performed on the primary endpoint when 30 participants have been randomised and completed 8 weeks of the intervention.. The interim-analysis will be performed by the PI, blinded for the treatment allocation. The results of the analysis will be discussed with the Safety Advisory Board and the research team in a joint meeting. A re-calculation of the total target sample will be applied based on the interim analysis, with any changes to final intended sample size reported to the ethics committees overseeing the conduct of the project.

### Ethics and dissemination

#### Research ethics approval

Ethical approval to conduct the clinical trial was granted by two Human Research Ethics Committees (HREC) accredited by the National Health and Medical Research Council (NHMRC): the University of Technology Sydney HREC (NHMRC Accreditation: #EC00146; HREC project approval: ETH17–1926) on the 15.02.2018, and then further approved by the Endeavour College of Natural Health HREC (NHMRC Accreditation: #EC00146; HREC project approval: 20,180,226) on the 26.02.2018.

#### Protocol amendments

The protocol was amended to expand the recruitment strategy to reach a wider target. The additional strategy was to approach Brisbane primary schools via email to advertise the trial through their social media or newsletters to the broader school community. This strategy was approved by both UTS and Endeavour HREC after each individual school principal gives permission and signs an approval form which is forwarded onto the UTS HREC.

#### Consent or assent

Explanation of the clinical trial and what participation involves initially occurs over the phone at the first point of contact when the parent of the child with NE contacts the researchers. After the parent reads the information sheet and discusses with the child and other family members the implications for the child participating, and then decide they would like to proceed with participation, they contact the researcher to determine the child’s eligibility. The researcher will use the universal screening tool to determine eligibility. If it is determined the participant is eligible, the researcher books a date and time with the parent for the baseline visit to Endeavour College of Natural Health, 269 Wickham Street, Fortitude Valley.

In accordance with Ethical Research Involving Children (ERIC) Charter [41], all visits throughout the study will occur in a room with a glass wall. At the initial visit final queries or discussion will occur as per parental requirements for details of the trial. The researcher will ask the child what is their understanding of what it means for them to participate in the trial to determine their capacity in giving assent. If the child does not have a clear understanding of the trial, the researcher will explain in language at a developmental level appropriate for the child. A child’s refusal to participate is respected if they have the capacity to give consent [42]. The formal consent will be signed after the following criteria have been met; the researcher is satisfied the parent and child have an individual understanding of the trial and are in agreement with each other without conflict in participation, and the Patient Information Sheet has been read thoroughly by the parent and they have had the opportunity to discuss it with their family and others. Even young children with limited cognitive capacity should be engaged at their level in discussion about the research and its likely outcomes [42]. A consent form for the parent/guardian of the participant to sign together with the Researcher’s signature is to be kept by the parent as their copy of the consent.

Participants will be provided with access to a reduced payment for car parking if they choose to drive to the college for the visits. The car parking ticket will be validated at the College which gives the participant a reduced parking fee at a local car park. The participants will also receive a fifty ($50) dollar voucher that can be used universally at any retail outlet with an EFTPOS or credit care facility at the final visit (Week 32) to the College. This is a small re-imbursement for the travel costs to the site for clinic visits.

#### Termination criteria

Discontinuation of the trial would only occur due to ethical implications, statistical, recruitment or safety concerns.

Ethical and Statistical implications include:

1. Adverse Events will be recorded and analysed to confirm if they are related to the intervention being trialled by a safety committee. If deemed more than 40% are due to the intervention, the trial will be terminated.

Safety concerns include:

1. If data or results indicate patient safety is at risk, the trial will be terminated.
2. If the severity or number of adverse events related to the intervention occur [> 40%], the trial will be terminated. An adverse event is defined as any untoward medical occurrence that results in hospitalisation or prolongation of hospitalisation, or is life-threatening, or results in death or disability, including birth defects [43].

Recruitment:

1. Insufficient accrual of participants (less than 30 people) within an 18-month time frame.

It is unlikely that this research project would be stopped unexpectedly unless due to unforeseen circumstances. This trial is being monitored by UTS HREC and Endeavour HREC. In addition, this is a TGA approved product and is already available for retail purchase in Australia and the United States of America. All safety data pertaining to the product is available, however, safety is still being monitored in this trial.

#### Confidentiality

Participant raw data will be collected on paper case report forms with a de-identified study code. Identification of the participant is through a single password-protected electronic file which matches name with study code. The Study code is only used on all data collection forms (paper and electronic). Data will all be analysed in summarised form. The electronic file which contains the identities of the participants is stored on restricted drive in the Endeavour College which is only accessible by the researchers. Only de-identified data analysed in summary form will be used in presentation or publication. Data (electronic and hard copy) will be stored for 7 years and then permanently destroyed.

#### Declaration of interests

This research project is funded through the University of Technology, Sydney by both; an Innovations Connection Grant, awarded by the Dept. of Industry, Innovation & Science (Federal Gov’t), and Seipel Group Pty Ltd. (ACN 152681028), PO Box 3449, Newmarket, 4051, Queensland, Australia. This research is being conducted by Endeavour College of Natural Health and sponsored in Australia by Seipel Group, PO Box 3449, Newmarket, 4051, Queensland, Australia.

#### Access to data

Access to raw data will only be available to research staff including the trial coordinator, the PI and CI. The contractual agreement states that the funding sponsor of the trial may not have access to any data, throughout or after the trial until all data is analysed and published. Therefore, allowing independent research to be conducted and the research staff blinded to allocation until analysis is completed.

#### Ancillary and post-trial care

At the completion of the trial (after the 6 months of post monitoring), each child will receive two bottles of the Bedtime Buddy. Due to the blinding of the trial until after analysis, this allows those who may be on the placebo to have access to the investigational product. If any participant suffers psychological harm during the trial, counselling services will be provided for the child.

#### Dissemination policy

Neither the complete nor any part of the results of the study carried out under this protocol, nor any of the information provided by the sponsor for the purposes of performing the study, will be published or passed on to any third party without the consent of the study sponsor. Any investigator involved with this study is obligated to provide the sponsor with complete test results and all data derived from the study.

## Discussion

This study examines a novel treatment for an under-researched health condition affecting many children. Despite the existing data supporting the plausibility of effectiveness for this treatment in this condition [30], the social and psychological impacts of nocturnal enuresis present challenges to conducting this study as planned. The recruitment of children in clinical trials is acknowledged as more difficult than adults for many reasons including consent by proxy, difficulty in identifying eligible participants, and higher required sample due to lower burden of disease [44]. Recruitment can be particularly problematic for a condition such as NE given only half of parents with children with NE consult a medical practitioner [16]. For researchers, the role of health professionals can be integral to identifying potential study participants for a clinical trial, and this is particularly pronounced in a paediatric study [44]. Given the perception of NE as a developmental condition among some parents [9], recruitment not only requires identifying potential participants through general parenting groups and social networks but also relies on parents acknowledging the social, psychological and quality of life impact of NE on their child.

The control method for our study may also impact on recruitment. While the placebo-controlled trial (PCT) is still considered the most robust method for testing new interventions the acceptability of placebo interventions for children is a contested issue [25]. Researchers and policy makers have increasingly acknowledged the value of comparative effectiveness (CE) studies in vulnerable populations rather than PCTs but CE trials rely on an established effective treatment for the active intervention to be compared against [25]. Certainly, CE trials are favoured by medical staff and parents when seeking consent [45]. Despite a number of widely accepted therapies [19] there is insufficient evidence to support such a comparative treatment for NE in children. For this reason, placebo-control is still appropriate for a non-life threatening condition such as NE [44] yet concerns about their child being randomized to ineffective treatments may outweigh the benefits for some parents [45]. Irrespective of these challenges, the available evidence for efficacy for the active intervention in a similar condition coupled with the absence of strong evidence for other effective treatments justifies the need for this study.

## Data Availability

Not applicable.

## Abbreviations

AE: Adverse event
ANOVA: Analysis of variance
CM: Complementary medicine
CRF: Case report form
ERIC: Ethical Research Involving Children
HREC: Human Research Ethics Committee
IIQ: Incontinence impact questionnaire
NE: Nocturnal enuresis
OAB: Overactive bladder
OR: Odds ratio
PinQ: Paediatric quality of life questionnaire
PNE: Primary nocturnal enuresis
RN: Registered nurse
SAE: Serious adverse event
UDI: Urinary distress inventory
UI: Urinary incontinence
UTI: Urinary tract infection
